# The impact of rescue or maintenance therapy with EGFR TKIs for Stage IIIb-IV non-squamous non-small-cell lung cancer patients requiring mechanical ventilation

**DOI:** 10.1186/1471-2253-14-55

**Published:** 2014-07-16

**Authors:** Te-Chun Hsia, Chih-Yen Tu, Hung-Jen Chen

**Affiliations:** 1 Division of Pulmonary and Critical Care Medicine, China Medical University Hospital, Taichung, Taiwan; 2 Department of Internal Medicine, China Medical University Hospital, Taichung, Taiwan; 3 Department of Respiratory Therapy, China Medical University, Taichung, Taiwan; 4 Department of Life Sciences, National Chung Hsing University, Taichung, Taiwan

**Keywords:** Stage IIIb-IV non-squamous NSCLC, Respiratory failure, EGFR TKI, SAPS II score, SOFA score

## Abstract

**Background:**

The toxicity of epidermal growth factor receptor (*EGFR*) tyrosine kinase inhibitors (TKIs) is less than that of cytotoxic agents. The reports of dramatic response and improvement in performance status with the use of *EGFR* TKIs may influence a physician’s decision-making for patients with non-squamous non-small cell lung cancer (NSCLC) and life-threatening respiratory distress. The aim of this study was to evaluate the outcome of rescue or maintenance therapy with *EGFR* TKI for stage IIIb-IV non-squamous NSCLC patients requiring mechanical ventilation.

**Methods:**

Eighty-three Asian patients with stage IIIb-IV non-squamous NSCLC and who required mechanical ventilation between June 2005 and January 2010 were evaluated.

**Results:**

Of the 83 patients, 16 (19%) were successfully weaned from the ventilator. The use of *EGFR* TKI as rescue or maintenance therapy during respiratory failure did not improve the rate of successful weaning (standard care 18% vs. with *EGFR* TKI, 22%; *p =* 0.81) in univariate and multivariate analyses.

**Conclusions:**

Rescue or maintenance therapy with *EGFR* TKI for stage IIIb-IV non-squamous NSCLC patients requiring mechanical ventilation was not associated with better outcome. An end-of-life discussion should be an important aspect in the care of this group of patients, since only 19% were successfully weaned from mechanical ventilation.

## Background

Lung cancer, especially adenocarcinoma, is the leading cause of cancer-related deaths in 2013 [1]. Despite advances in therapy, the five-year survival for stage IIIb-IV patients with metastatic lung cancer is around 2-22% [2]. Nonetheless, patients with lung cancer sometimes accept mechanical ventilation (MV) support [3-8] because many are unaware of the significant risk of death [9].

Epidermal growth factor receptor (*EGFR*) is expressed in a large proportion of non-small-cell lung cancer (NSCLC) tumors [10]. Two *EGFR* mutations (exon 19 deletion and exon 21 L858R substitution) that cluster around the adenosine-5′-triphosphate-binding pocket of the *EGFR* tyrosine kinase (TK) domain are highly responsive to *EGFR* TK inhibitors (TKIs) like gefitinib or erlotinib [11]. Phase III trials comparing chemotherapy to gefitinib as first-line treatment for advanced NSCLC patients with *EGFR*-activating mutations have shown that gefitinib significantly improves progression-free survival [12-14]. When samples cannot be enriched for *EGFR* mutation analysis, never-smokers and Asian non-squamous NSCLC patients are associated with *EGFR* mutations and *EGFR* TKIs responses [15].

Acquired resistance to *EGFR* TKIs develops in 9.7-13.3 months in patients with *EGFR* mutations [16-18]. Because the toxicity of *EGFR* TKIs is less than that of cytotoxic agents, their use for patients with non-squamous NSCLC and poor performance status (PS) has also been proven [19,20].

Lung cancer patients with respiratory failure have extremely poor PS. As reported, dramatic response [21] and improvement in PS [19] with the use of *EGFR* TKIs may influence a physician’s decision-making for patients with non-squamous NSCLC and life-threatening respiratory distress. Lung cancer patients who are ventilator-dependent consume considerable resources but have low quality of life in their remaining years. Rescue or maintenance *EGFR* TKIs can induce apoptosis of lung cancer cells and may favor MV weaning for critical non-squamous NSCLC patients. The objective of this study was to assess the MV weaning rate and outcome of rescue or maintenance therapy with *EGFR* TKIs for stage IIIb-IV non-squamous NSCLC in Asian patients requiring MV. To date, the present study is first to address this issue.

## Methods

### Patient identification

Lung cancer patients from China Medical University Hospital, a 2000-bed medical center and teaching hospital for referred patients in Taiwan, between June 2005 and January 2010 were included. The hospital’s institutional review board approved the study protocol (DMR99-IRB0149) and consent was waived because of the retrospective design. The medical records of 205 lung cancer patients placed on MV because of life-threatening respiratory failure were analyzed. As a care policy in the study hospital, patients who needed MV >24 hours had to be admitted to the intensive care unit (ICU).

Life-threatening respiratory failure was defined as retention of carbon dioxide, hypoxemia, or evidence of respiratory muscle fatigue. Hospice care was defined as a patient refusing any aggressive treatment after endotracheal tube insertion. In case of recurrent respiratory failure requiring MV, only the first was considered. “Ventilator-dependent” was defined as a patient needing MV more than 100 days. In Taiwan, stabilized (ICU) patients needing MV care for more than 21 days are transferred to a respiratory care center. Patients who still require MV with stable condition are subsequently discharged from the hospital and transferred to the chronic respiratory care ward. In this series, no patient transferred to the chronic respiratory care ward since those who required MV more than 100 days was weaned from MV. As such, “ventilator-dependent more than 100 days” and “non-survivors” were combined into the same group for analysis.

**Figure 1:**
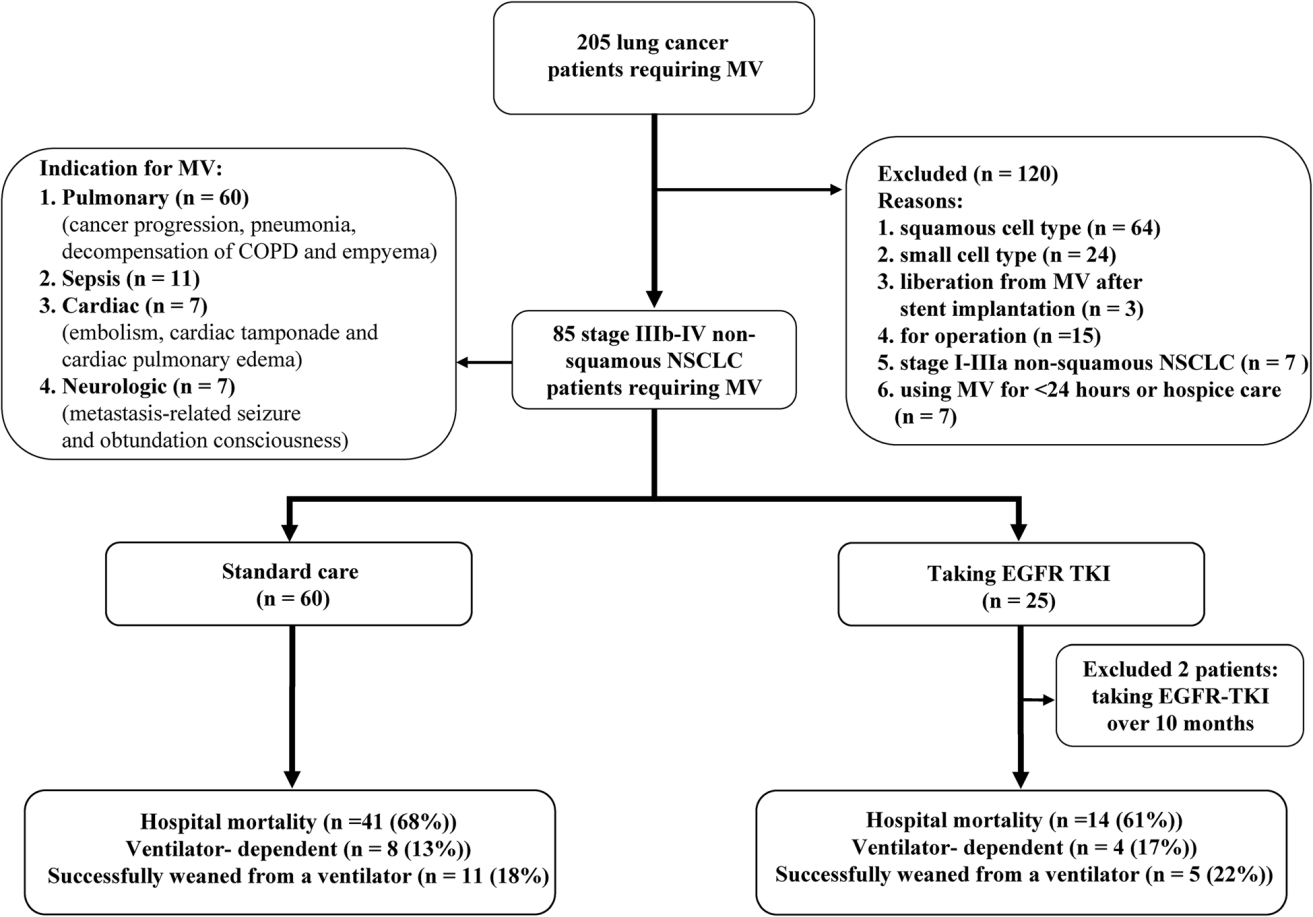
Flow chart of the study and selection of participants.

Based on the inclusion and exclusion criteria (Figure 1), patients accepting stent implantation for obstructive tumors [22], those who used MV for surgery, and those who used MV for <24 hours or hospice care were all excluded to reduce confounding factors. Patients taking gefitinib or erlotinib over 10 months were also excluded from the maintenance therapy group because the possibility of acquired resistance to *EGFR* TKI could not be ruled out [16-18].

### Data collection and definitions

Demographic, physiologic, and clinical data, including age, sex, smoking history, co-morbidities, and main indication for MV, were collected. The cancer disease characteristics included sub-type, extent of use of the TNM 7th Edition of the Lung Cancer Stage Classification System [2], PS within the preceding week (Eastern Cooperative Oncology Group scale, ECOG-PS) [23], and specific treatments administered before respiratory failure (e.g. surgery, *EGFR* TKI target therapy, chemotherapy, and/or radiation therapy). The presence of metastasis was also recorded, whether already known or evidenced within the period of ventilator support. The cancer disease status was assessed as controlled or non-controlled (cancer disease progression). Patients with “newly-diagnosed lung cancer” in a non-controlled status were defined as those waiting for a decision to treat or had been treated with less than two cycles of chemotherapy or less than 28 days of *EGFR* TKI.

Laboratory data obtained within 24 hours of ventilatory support were collected. These included hemoglobin, white blood cell count, platelet count, coagulation profile, levels of blood urea nitrogen, creatinine, and electrolytes, liver function tests, arterial blood gas measurements, and blood cultures.

The severity of the acute illness was assessed using the Acute Physiology and Chronic Health Evaluation (APACHE) II score [24], Simplified Acute Physiology Score (SAPS) II [25], and Sequential Organ Failure Assessment (SOFA) [26] based on data collected within 24 hours of MV. Severe sepsis/septic shock was diagnosed using the definitions of the American College of Chest Physicians/Society of Critical Care Medicine Consensus Conferences [27].

All of the patients were evaluated longitudinally to determine their hospital outcomes. Furthermore, the administration of specific anti-cancer treatments after discharge, i.e., chemotherapy, radiation therapy, and *EGFR* TKI target therapy, as well as vital status at 18 months, were recorded.

The *EGFR* TKIs group was defined as patients with non-squamous NSCLC and respiratory failure treated based on the general principles of critical care and rescue/maintenance *EGFR* TKIs. The standard care group was defined as patients with non-squamous NSCLC and respiratory failure treated based on the general principles of critical care without radiotherapy, chemotherapy, or *EGFR* TKIs.

### EGFR mutation test

Most tumor samples were obtained from paraffin-embedded blocks made on initial diagnosis. The DNA sequences of exons 19 and 21 of *EGFR* were determined by direct forward and reverse sequencing of the polymerase chain reaction (PCR) product from nested PCR reactions, as described previously [28].

### Statistical analysis

Continuous variables were reported as median (inter-quartile range, IQR) and categorical variables as number (percentage). The SAPS II and the SOFA scores were expressed in points while *p* values were calculated using two-sided chi-square and Fisher’s exact tests for categorical variables and the Mann–Whitney *U* test for continuous variables. The curves of ventilator weaning were obtained using the Kaplan-Meier method and compared using the Log-Rank test.

Univariate and multivariate analyses were performed to identify factors associated with weaning from MV. Variables selected by univariate analysis (*p* ≤ 0.5) and those considered as clinically relevant (age) were entered into a logistic regression model. Results were expressed as odds ratios (ORs), with their 95% confidence intervals (95% CIs). Statistical significance was set at *p* < 0.05. All statistical analyses were performed using the SPSS software, version 17.0 (SPSS Inc., Chicago, IL, USA).

## Results

Of 205 potentially eligible patients with lung cancer requiring MV, 64 with squamous cell type, 24 with small cell type, 3 with liberation from MV after stent implantation, 15 who used MV for operation, seven with stage I-IIIa non-squamous NSCLC, seven who used MV for <24 hours or hospice care, and two taking *EGFR* TKIs over 10 months were excluded. The resulting sample included 83 stage IIIb-IV non-squamous NSCLC Asian patients with life-threatening respiratory failure. Twenty-three 23 (28%) used gefitinib or erlotinib for rescue or maintenance therapy and 60 (72%) had standard care (Figure 1). Their baseline clinical characteristics were summarized in Table 1.The main reasons for MV were pulmonary problems (e.g. lung cancer progression, pneumonia, decompensation of chronic obstructive pulmonary disease, and empyema) (n = 58; 70%), sepsis (n = 11; 13%), cardiovascular disease (i.e. embolism, cardiac tamponade, cardiac pulmonary edema) (n = 7; 8%), and neurologic problems (i.e. metastasis-related seizure and obtunded consciousness) (n = 7; 8%). Some patients had more than one reason for MV (Figure 1).

**Table 1:** Characteristics and prognosis of stage IIIb-IV non-squamous NSCLC patients requiring mechanical ventilation with standard care or EGFR TKIs for rescue therapy

**Variables**	**All Patients**	**Standard Care**	**EGFR TKIs**	***p*-value**
**Subjects**	83	60	23	
**Age yrs**	68.0 (17)	68.5 (18)	68.0 (15)	0.84
**Gender**				
Female	36 (43)	24 (40)	12 (52)	0.45
Male	47 (57)	36 (60)	11 (48)	
**Smoking**	34 (41)	25 (42)	9 (39)	1.00
**Co-morbidities**				
No	43 (52)	32 (53)	11 (48)	0.84
Yes	40 (48)	28 (47)	12 (52)	
**ECOG-PS**				
0-2	26 (31)	17 (28)	9 (39)	0.49
3-4	57 (69)	43 (72)	14 (61)	
**Type of lung cancer**				
Adenocarcinoma	76 (92)	55 (92)	21 (91)	1.00
Large cell	7 (8)	5 (8)	2 (9)	
**Cancer status**				
Controlled	11 (13)	7 (12)	4 (17)	0.76
Uncontrolled, newly diagnosed	36 (43)	27 (45)	9 (39)	
Uncontrolled, progression	36 (43)	26 (43)	10 (44)	
**Treatment before respiratory failure**				
Combined therapy	24 (29)	18 (30)	6 (26)	0.26
EGFR TKI only	9 (11)	4 (7)	5 (22)	
Chemotherapy only	31 (37)	23 (38)	8 (35)	
No treatment	19 (23)	15 (25)	4 (17)	
**Indication for MV**				
Pulmonary	58 (70)	41 (68)	17 (74)	0.90
Sepsis	11 (13)	9 (15)	2 (9)	
Cardiac	7 (8)	5 (8)	2 (9)	
Neurologic	7 (8)	5 (8)	2 (9)	
**APACHE II score, points**	23.0 (9)	23.5 (11)	23.0 (9)	0.86
**SAPS II score, points**	54.0 (19)	54.5 (19)	54.0 (23)	0.84
**SOFA score, points**	7.0 (5)	7.0 (4)	7.0 (5)	0.70
**Use of vasopressors**	39 (47)	29 (48)	10 (44)	0.88
**Positive blood culture**	15 (18)	12 (20)	3 (13)	0.54
**Outcomes**				
Hospital mortality	55 (66)	41 (68)	14 (61)	0.81
Ventilator- dependent > 100 days	12 (15)	8 (13)	4 (17)	
Successfully weaned from a ventilator	16 (19)	11 (18)	5 (22)	

Data are median (inter-quartile range) for quantitative data and number (%) for qualitative data.

The *p* values were calculated by two-sided chi-square and Fisher’s exact tests for categorical variables and by the Mann–Whitney *U* test for continuous variables.

Abbreviations: *APACHE*, Acute Physiology and Chronic Health Evaluation; *ECOG-PS*, Eastern Cooperative Oncology Group scale, Performance status; *EGFR* TKIs, Epidermal growth factor receptor tyrosine kinase inhibitor; *MV*, Mechanical ventilation; *SAPS*, Simplified Acute Physiology Score; *SOFA*, Sequential Organ Failure Assessment.

Sixteen patients (19%) were successfully weaned from the ventilator and 67 (81%) remained ventilator-dependent or died (Figure 1). No patient was transferred to the chronic respiratory care ward since those who required MV more than 100 days were successfully weaned from MV. Eight of the 16 patients successfully weaned were discharged from the hospital and received specific anti-cancer treatments. Two patients received chemotherapy and *EGFR* TKIs, three had chemotherapy, and three had *EGFR* TKIs only. All of the patients died within 415 days.

**Figure 2:**
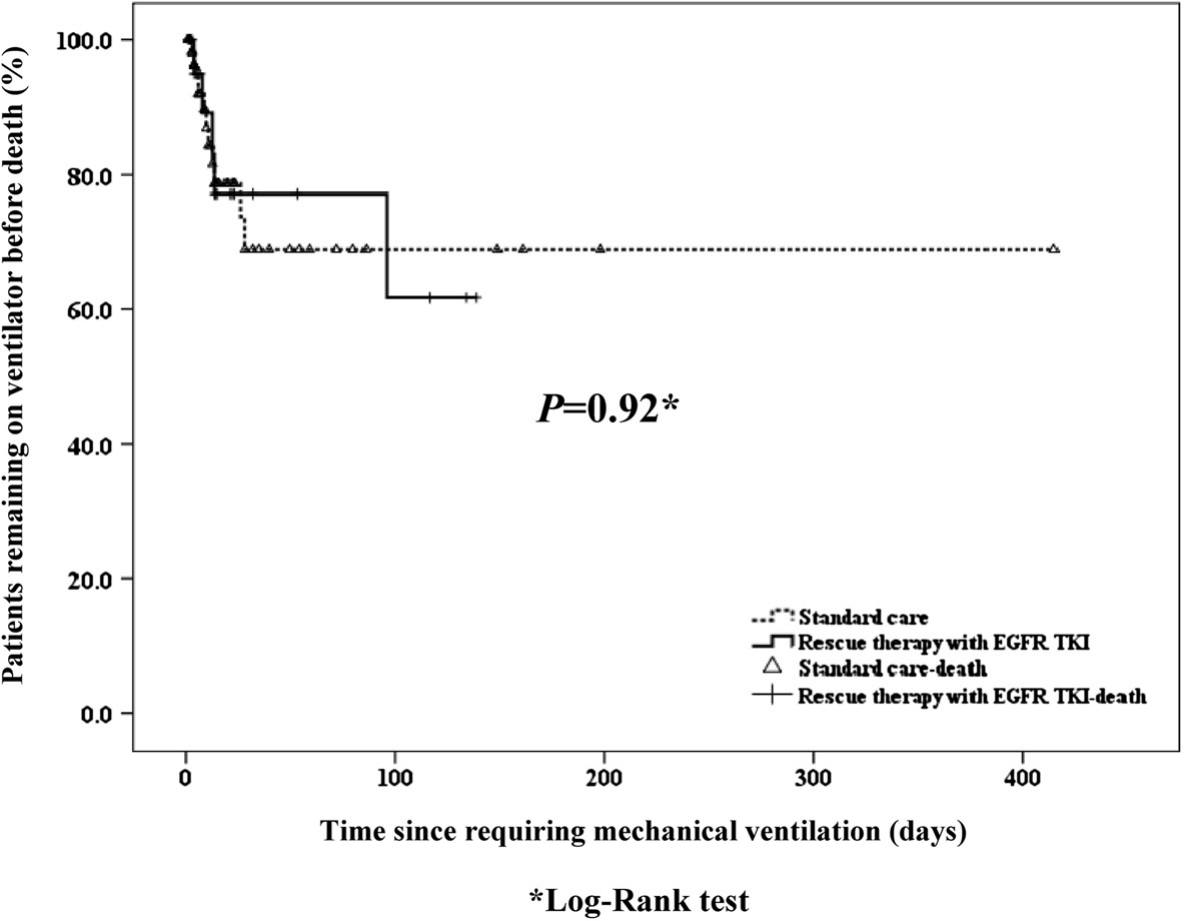
**Kaplan-Meier curves of successful weaning from the ventilator between standard care patients and those taking EGFR TKIs for maintenance or rescue therapy.** *Log-rank test; EGFR TKIs, Epidermal growth factor receptor tyrosine kinase inhibitors.

There were no significant differences in baseline clinical characteristics, including APACHE II, SAPS II, and SOFA scores between the standard care patients and those who took gefitinib or erlotinib as rescue or maintenance therapy during the period of respiratory failure. There was no significant difference in outcomes (hospital mortality, ventilator-dependence >100 days, and successful weaning) between the two groups (Table 1). Kaplan-Meier curves of successful weaning from MV with Log-Rank test also revealed no significant differences (*p* = 0.92) (Figure 2).

The main laboratory and physiologic variables were recorded and compared by univariate and multivariate analysis between “ventilator-dependent >100 days/non-survivors” and “successfully weaned from the ventilator” during the period of respiratory failure (Table 2). Variables that differed significantly were worse severity scores (SOFA or SAPS II scores), not taking *EGFR* TKIs.

**Table 2:** Predicting successful weaning from mechanical ventilation

**Variables**	**Ventilator-dependent >100 days or non-survivors**	**Weaned from the ventilator**	**Univariate Analysis OR (95% CI)**	**Multivariate Analysis (Mode With SOFA) OR (95% CI)**	**Multivariate Analysis (Mode With SAPS II) OR (95% CI)**
**Subjects**	67	16			
**Age yrs**	68.0 (16)	71.5 (21)	1.01 (0.96-1.05)	1.01 (0.95-1.07)	1.03 (0.97-1.10)
**Gender**					
Female	27 (40)	9 (56)	1.91 (0.63-5.73)	1.62 (0.34-7.85)	1.22 (0.27-5.59)
Male	40 (60)	7 (44)			
**Smoking**	29 (43)	5 (31)	0.60 (0.19-1.90)	1.27 (0.23-7.05)	0.83 (0.16-4.32)
**Co-morbidities**					
No	34 (51)	9 (56)	0.80 (0.27-2.40)	-	-
Yes	33 (49)	7 (43)			
**ECOG-PS**					
0-2	20 (30)	6 (38)	0.71 (0.23-2.22)	-	-
3-4	47 (70)	10 (63)			
**Type of lung cancer**					
Adenocarcinoma	60 (90)	16 (100)	**-**^**+**^	**-**	**-**
Large cell	7 (10)	0			
**Cancer status**					
Controlled	9 (13)	2 (13)	1.29 (0.23-7.19)	-	-
Uncontrolled, newly diagnosed	30 (45)	6 (38)			
Uncontrolled, progression	28 (42)	8 (50)			
**Treatment before respiratory failure**					
Combined therapy	21 (31)	3 (19)	2.04 (0.47-8.91)	2.18 (0.45-10.53)	1.70 (0.36-7.99)
EGFR TKI only	6 (9)	3 (19)			
Chemotherapy only	24 (36)	7 (44)			
No treatment	16 (24)	3 (19)			
**Indication for MV**					
Pulmonary	48 (72)	10 (63)	0.80 (0.09-7.40)	-	-
Sepsis	9 (13)	2 (13)			
Cardiac	6 (9)	1 (6)			
Neurologic	4 (6)	3 (19)			
**APACHE II score, points**	25.0 (12)	21.0 (7)	0.91 (0.82-1.00)	-	-
**SAPS II score, points**	55.0 (23)	46.5 (15)	0.94 (0.89-0.99)^*^	-	0.92 (0.86-0.99)^*^
**SOFA score, points**	8.0 (4)	6.0 (4)	0.80 (0.65-0.98)^*^	0.66 (0.46-0.94)^*^	-
**Use of vasopressors**	33 (49)	6 (38)	0.62 (0.20-1.89)	3.77 (0.57-25.07)	1.35 (0.33-5.50)
**Positive blood culture**	13 (19)	2 (13)	0.59 (0.12-2.94)	-	-
**Taking EGFR TKI**	18 (27)	5 (31)	1.24 (0.38-4.06)	0.98 (0.26-3.77)	1.14 (0.30-4.27)

Data are median (inter-quartile range) for quantitative data and number (%) for qualitative data.

The *p* values were calculated by two-sided chi-square and Fisher’s exact tests for categorical variables and by the Mann–Whitney *U* test for continuous variables. OR, odds ratio (See Tables 1 for expansion of other abbreviations).

^+^Difficult check due to statistical convergence problem.

^*^p < 0.05.

Eight (35%) of 23 patients taking *EGFR* TKIs for maintenance or rescue therapy had samples enriched for *EGFR* mutation analysis. Three patients had exon 19 deletions, three had L858R, one had double mutation of exon19 deletions and L858R, and one was wild-type. Patients who were never-smokers and/or positive for *EGFR* mutation accounted for 72% of those who were “ventilator-dependent >100 days or non-survivors” (Table 3). Statistical analysis was not performed in Table 3 because the sample size was too small.

**Table 3:** EGFR mutation, non-smoking percentage, and SAPS II and SOFA scores of patients taking EGFR TKIs for rescue/maintenance therapy (n = 23)

**Variables**	**Ventilator-dependent >100 days or non-survivors**	**Weaned from the ventilator**
**Subjects**	18	5
**EGFR mutation status of 8 samples**	6 positive	1 positive
	1 negative	
*Smoking	4	1
**Nonsmoking**	7	3
**Positive EGFR mutation + nonsmoking**	13 (72)	4 (80)
**SAPS II score**	54.0 (23.0)	46.0 (20.5)
**SOFA score**	8.0 (4.5)	5.0 (3.5)

*Excluding patients receiving EGFR mutation test.

Data are median (inter-quartile range) for quantitative data and number (%) for qualitative data.

See Tables 1 for abbreviations.

## Discussion

To date, this study is the largest investigation on stage IIIb-IV non-squamous NSCLC patients requiring MV. This study is also the first to review rescue or maintenance therapy with *EGFR* TKIs in non-squamous NSCLC patients with respiratory failure. Based on the findings, rescue or maintenance therapy with *EGFR* TKIs for stage IIIb-IV non-squamous NSCLC patients requiring MV is not associated with better outcome.

When acute life-threatening respiratory failure develops in a patient with lung cancer, physicians are doubtful about the wisdom of endotracheal intubation with MV support. This is because lung cancer patients have a special condition in which tumor extension during treatment of reversible problems may preclude successful weaning from MV. Although “do not resuscitate” is recommended by physicians for critically ill lung cancer patients, patients also want to try chemotherapy [29] or new anti-cancer medications [30] and have accepted MV support [3-8].

The results reveal that 69% of patients with ECOG-PS 3 or 4 are admitted for MV (Table 1), suggesting a rather poor outcome, with successful weaning rates of 19% for stage IIIb-IV non-squamous NSCLC patients, similar to those of studies by Reiner et al. [5] and Soares et al. [7]. Only eight (10%), or half of the surviving patients discharged from the hospital, have received specific anti-cancer treatments.

In this series, the “ventilator-dependent >100 days” and “non-survivors” have been combined into the same group because their prognoses preclude performing endotracheal intubation (Table 2). Although individual outcome may be difficult to predict, those with lower SAPS II (*p* = 0.03) or SOFA (*p* = 0.02) scores have higher rates of weaning from the ventilator. These results here are similar to those of Toffart et al. [31] and Roques et al. [6].

Undoubtedly, gefitinib and erlotinib have powerful anti-tumor activity and are superior to chemotherapy in patients with advanced non-squamous NSCLC with *EGFR* mutations [14,17]. However, despite similar baseline clinical characteristics in the study groups during the period of respiratory failure, intake of *EGFR* TKIs as rescue or maintenance therapy does not lead to a better rate of weaning from the MV (Table 1). This is because the severity of acute illness scores or organ dysfunction has a larger impact on the prognosis of critically ill lung cancer patients than tumor-related factors [31]. In 23 patients taking *EGFR* TKIs for rescue/maintenance therapy, the “ventilator-dependent >100 days/non-survivors” group also has higher SAPS II and SOFA scores, which corroborate this (Table 3).

The current study has several limitations. First, it is a single-center study, so the generalizability of the results to other hospitals is unknown. Second, this is a retrospective study. The sample size of the group taking *EGFR* TKIs for rescue or maintenance therapy is small. However, a large prospective, randomized trial to confirm the results in terms of maintenance or rescue therapy with gefitinib or erlotinib for stage IIIb-IV non-squamous NSCLC patients requiring MV will probably not be feasible. Third, only 35% (8/23) of samples were enriched for *EGFR* mutation analysis. Otherwise, the percentage of never-smokers and/or those positive for *EGFR* mutation was 72% in the “ventilator-dependent >100 days/non-survivors” group. The high percentage (72%) against the thought of poor outcome is related to ineffective *EGFR* TKI.

Why did 29% of patients receive *EGFR* TKI for rescue or maintenance therapy but others did not? The main reason may be that clinicians expect *EGFR* TKIs to be effective by the status of never-smokers and non-squamous NSCLC Asian patients [15]. In the study hospital, *EGFR* mutation could not be checked until 2009. In the standard care group, 20% of patients stopped using *EGFR* TKIs after respiratory failure because it was ineffective or because of interstitial pneumonitis. With a high selection for *EGFR* TKI treatment, the use of *EGFR* TKIs does not lead to better outcomes if the patient’s acute severity score reaches a critical level.

## Conclusions

Rescue or maintenance therapy with *EGFR* TKI for stage IIIb-IV non-squamous NSCLC patients requiring MV was not associated with better outcome. An end-of-life discussion should be an important aspect in the care of this group of patients since only 19% were successfully weaned from MV.

## Abbreviations

APACHE: Acute physiology and chronic health evaluation; ECOG-PS: Eastern cooperative oncology group scale, performance status; EGFR TKIs: Epidermal growth factor receptor tyrosine kinase inhibitor; ICU: Intensive care unit; MV: Mechanical ventilation; NSCLC: Non-small cell lung cancer; SAPS: Simplified acute physiology score; SOFA: Sequential organ failure assessment; COPD: Chronic obstructive pulmonary disease.

## Competing interests

The authors declare that they have no competing interests

## Authors’ contributions

TCH, CYT and HJC designed the study and interpreted the results. HJC drafted the manuscript. All authors edited and approved the final manuscript.

## Pre-publication history

The pre-publication history for this paper can be accessed here:

http://www.biomedcentral.com/1471-2253/14/55/prepub
